# New-Onset POAF After Craniotomy: Impact on Neurological Outcome, ICU Utilization, and Mortality

**DOI:** 10.3390/jcm15082959

**Published:** 2026-04-13

**Authors:** Obayda Azizy, Ahmad Alwakaa, Mussab Alali, Mohamad Amer Nashtar, Mortimer Gierthmuehlen, Omar Alwakaa, Ali Canbay, Niklas Thon, Polykarpos Christos Patsalis

**Affiliations:** 1 Ruhr University Bochum, Division of Cardiology, Angiology and Internal Emergency Medicine, Department of Medicine, Knappschaft Kliniken University Hospital Bochum, 44892 Bochum, Germany; mohamad.nashtar@knappschaft-kliniken.de (M.A.N.); polykarpos.patsalis@knappschaft-kliniken.de (P.C.P.); 2 Hannover Medical School, 30625 Hannover, Germany; ahmad.alwakaa@stud.mh-hannover.de; 3 Ruhr University Bochum, 44892 Bochum, Germany; musaab.alali@edu.ruhr-uni-bochum.de; 4 Ruhr University Bochum, Department of Neurosurgery, Knappschaft Kliniken University Hospital Bochum, 44892 Bochum, Germany; mortimer.gierthmuehlen@knappschaft-kliniken.de (M.G.); niklas.thon@knappschaft-kliniken.de (N.T.); 5 Harvard Medical School, Boston, MA 02115, USA; oalwakka@bidmc.harvard.edu; 6 Ruhr University Bochum, Department of Medicine, Knappschaft Kliniken University Hospital Bochum, 44892 Bochum, Germany; ali.canbay@knappschaft-kliniken.de

**Keywords:** atrial fibrillation, postoperative atrial fibrillation, neurosurgery, craniotomy, perioperative complications, intensive care, brain–heart interaction

## Abstract

**Background/Objectives:** Atrial fibrillation is the most common sustained arrhythmia and frequently occurs in the perioperative setting. However, its clinical relevance in neurosurgical patients remains poorly defined despite increased vulnerability related to brain injury, inflammation, and perioperative stress. This study aimed to determine whether newly detected postoperative atrial fibrillation (POAF) identifies a higher-risk profile and is associated with postoperative complications, resource utilization, and short-term mortality compared with patients remaining in sinus rhythm (SR). **Methods:** We conducted a single-center retrospective cohort study (October 2020–April 2025). Among 2619 neurosurgical procedures, only patients with both pre- and in-hospital post-procedure ECGs and no pre-existing arrhythmia were included. POAF was defined as atrial fibrillation detected on any post-procedure ECG. Outcomes were compared using Welch’s *t*-test, χ^2^/Fisher’s exact tests, and odds ratios (OR) with 95% confidence intervals (CI). This study was not designed to estimate the incidence of POAF but rather to compare outcomes within a selected ECG-screened subgroup. **Results:** A total of 171 patients met the inclusion criteria: 79 (46.2%) developed POAF and 92 (53.8%) remained in SR. Patients with POAF were older and had a higher burden of cardiometabolic comorbidities and were more likely to undergo craniotomy/trepanation and emergency procedures. Compared with SR, POAF was associated with higher rates of postoperative complications, longer ICU and hospital stay, lower likelihood of discharge home, and higher short-term mortality. These findings reflect a selected ECG-screened cohort and should not be interpreted as the incidence of POAF in the overall neurosurgical population. **Conclusions:** Newly detected POAF is associated with a higher-risk postoperative profile in neurosurgical patients. It clusters with greater comorbidity burden, more invasive and urgent procedures, increased complications, prolonged hospitalization, reduced likelihood of discharge home, and higher short-term mortality. These findings support further evaluation of rhythm surveillance and perioperative management strategies in higher-risk neurosurgical populations.

## 1. Introduction

Postoperative atrial fibrillation (POAF) is a clinically important perioperative arrhythmia with relevant implications for patient outcomes across healthcare settings. Several studies have shown that POAF is associated with an increased long-term risk of ischemic stroke and thromboembolism after non-cardiac surgery [1,2]. In cardiac surgery populations, POAF is further linked to increased mortality and prolonged hospitalization [3,4]. Moreover, new-onset atrial fibrillation in critically ill patients is considered a marker of systemic stress and is associated with hemodynamic instability and adverse outcomes rather than a benign phenomenon [5].

Current clinical guidelines emphasize risk-based anticoagulation strategies rather than relying solely on arrhythmia duration [6,7,8,9]. While randomized data from stroke populations suggest that early anticoagulation can be safely implemented under structured conditions, these findings cannot be directly extrapolated to surgical populations due to substantially higher bleeding risks [10]. Mechanistically, perioperative atrial fibrillation has been linked to autonomic imbalance, inflammatory activation, and neurohumoral pathways, with particular involvement of central autonomic networks such as the insula [11,12,13,14]. In neurosurgical patients, additional factors including surgical invasiveness (e.g., craniotomy) and postoperative complications such as pneumonia may further increase physiological stress and arrhythmia susceptibility [15].

Despite the growing body of evidence on atrial fibrillation, data specific to neurosurgical populations remain limited. Patients undergoing intracranial surgery are frequently excluded from clinical trials or analyzed within heterogeneous non-cardiac surgical cohorts [2,3,4,16]. Furthermore, although extended rhythm monitoring has been shown to improve AF detection in stroke populations, the optimal monitoring strategy following neurosurgical procedures—including duration, intensity, and patient selection—remains unclear [17,18,19,20,21].

Therefore, this study aimed to evaluate whether newly detected postoperative atrial fibrillation identifies a higher-risk postoperative profile in neurosurgical patients and to assess its association with complications, resource utilization, and short-term mortality.

## 2. Methods

### 2.1. Study Design and Setting

Given the retrospective design and the use of fully anonymized clinical data collected during routine care, formal ethical approval was waived in accordance with institutional policy and national regulations. We conducted a single-center retrospective study at the Department of Neurosurgery, University Hospital Knappschaftskrankenhaus Bochum, Germany, a tertiary referral center for complex neurosurgical cases. We screened neurosurgical patients between October 2020 and April 2025. We excluded patients who were managed conservatively (no surgery), those missing either pre-procedure or post-procedure ECGs during hospitalization, and those with diagnosed arrhythmia prior to surgery. We included patients with sinus rhythm (SR) on a 12-lead ECG before the neurosurgical procedure and with at least one additional ECG obtained during hospitalization after surgery. Patients who developed atrial fibrillation on a post-procedure ECG during hospitalization were classified as POAF, while patients with SR on both pre- and post-procedure ECGs were classified as SR. Other supraventricular arrhythmias, including atrial flutter, were not classified as POAF. Due to the retrospective design, detailed information on episode duration and rhythm burden was not uniformly available. Postoperative rhythm surveillance was not standardized. Continuous telemetry was used according to routine clinical care in selected patients, particularly in higher-acuity or ICU settings, but not uniformly across the study population. Therefore, POAF detection relied on clinically obtained ECGs and may underestimate transient or asymptomatic episodes. ECG acquisition was not protocol-driven. Pre-procedure ECGs were generally obtained as part of routine perioperative assessment, whereas post-procedure ECGs were performed according to clinical indication during hospitalization, including symptoms, hemodynamic instability, cardiovascular risk profile, older age, or postoperative complications.

### 2.2. Data Collection

We collected demographic data including age, sex, height, weight, and body mass index (BMI). Documented comorbidities included hypertension, coronary artery disease, heart failure, diabetes mellitus, prior myocardial infarction, chronic obstructive pulmonary disease (COPD), dyslipidemia, peripheral arterial disease, and obesity. Surgical variables included primary diagnosis, surgical access (craniotomy/trepanation, burr-hole/twist-drill, or other), elective or emergency status, operative time, and intraoperative complications. Postoperative complications included pneumonia, fever, new neurological deficits, seizures, delirium, deep cranial infection or meningitis, cardiogenic shock, and re-operation. Laboratory values were analyzed before and after surgery, including hemoglobin, creatinine, INR, aPTT, CRP, troponin, and NT-proBNP. Resource utilization was assessed by ICU and total hospital length of stay, discharge destination (home, rehabilitation, oncologic/geriatrics ward, or nursing facility), and 30- and 90-day mortality.

## 3. Statistical Analysis

Continuous variables were compared using Welch’s *t*-test, and categorical variables were summarized as counts and percentages and compared using χ^2^ tests with Yates’ correction when appropriate. Fisher’s exact test was applied when expected cell counts were <5. For variables with more than two categories, global Fisher’s exact tests were performed, followed by pairwise comparisons with Benjamini–Hochberg adjustment for multiple testing. Odds ratios (OR) with 95% confidence intervals (CI) were calculated for binary outcomes. Statistical significance was defined as a two-sided α of 0.05. All analyses were performed using R (version 4.4.2). Due to the limited sample size and low number of events for several outcomes, multivariable regression analyses were not performed, as such models would likely be unstable and prone to overfitting. Therefore, the analyses should be considered exploratory and hypothesis-generating. Analyses were performed using available-case data. Denominators are reported where applicable and may vary slightly across variables due to missing data.

## 4. Results

**Figure 1:**
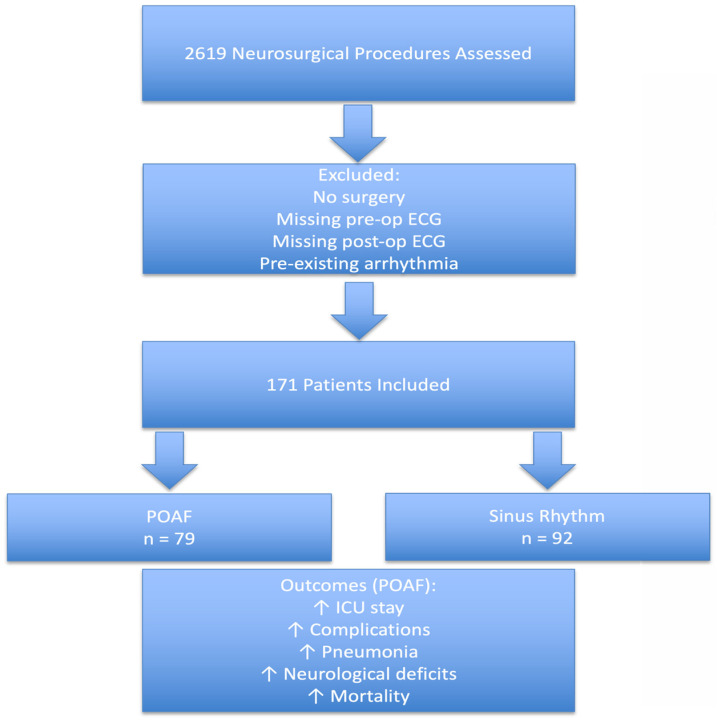
Study flowchart of patient selection and outcomes. A total of 2619 patients were screened, and 171 were included in the final analysis. Among these, 79 developed POAF and 92 remained in sinus rhythm.

Between October 2020 and April 2025, 2619 patients underwent neurosurgical treatment at the Department of Neurosurgery, University Hospital Knappschaftskrankenhaus Bochum, Germany. The patient selection process is illustrated in Figure 1. A total of 171 patients met the inclusion criteria and were included in the analysis. Among these, 79 patients (46.2%) developed newly detected POAF and 92 patients (53.8%) remained in sinus rhythm (SR). This proportion reflects the selected ECG-screened study cohort and should not be interpreted as the incidence of POAF in the overall neurosurgical population.

### 4.1. Baseline Characteristics

Baseline characteristics and comorbidities are summarized in Table 1 and Table 2. Patients with POAF were significantly older than those in the SR group (70.5 ± 14.7 vs. 63.9 ± 14.8 years; mean difference 6.7 years; *p* = 0.0037). Sex distribution did not differ significantly (male 44 [55.7%] vs. 44 [47.8%]; *p* = 0.27), nor did height, weight, or BMI. Patients with POAF had a more adverse cardiometabolic profile. Hypertension was more prevalent in the POAF group (73.4% vs. 40.2%; OR 4.07, 95% CI 2.05–8.32; *p* < 0.01), as was coronary artery disease (22.8% vs. 9.8%; OR 2.71, 95% CI 1.07–7.33; *p* = 0.022). Obesity was also more frequent (39.2% vs. 22.8%; *p* = 0.031). Heart failure was numerically higher in the POAF group but did not reach statistical significance (11.4% vs. 3.3%; *p* = 0.068). Other comorbidities did not differ significantly between groups.

### 4.2. Resource Utilization and Outcomes

Resource utilization and discharge outcomes are summarized in Table 3 and Table 4. Patients with POAF had longer ICU stays (3.84 vs. 2.16 days; mean difference 1.67 days; *p* = 0.015) and longer total hospital stays (14.24 vs. 10.86 days; mean difference 3.38 days; *p* = 0.016). Patients with POAF were less likely to be discharged home (45.8% vs. 66.3%; OR 0.43, 95% CI 0.22–0.85; BH-adjusted *p* = 0.044) and more likely to require rehabilitation or transfer to other care facilities. Short-term mortality was higher in the POAF group, with 30-day mortality of 8.9% compared to 1.1% in SR (OR 8.75, 95% CI 1.09–402.22; *p* = 0.025). Mortality at 60 and 90 days was unchanged from 30-day mortality. Mortality outcomes are also summarized in Table 1.

### 4.3. Surgical Characteristics

Surgical characteristics and operative variables are summarized in Table 5, Table 6 and Table 7. The distribution of primary diagnoses differed between groups (global Fisher’s exact test *p* = 0.018), with vascular cases more frequent in POAF (31.6% vs. 19.6%) and neoplastic cases less frequent (51.9% vs. 64.1%). However, no individual category remained significant after Benjamini–Hochberg adjustment. Surgical access showed a non-significant global trend (*p* = 0.079). In adjusted pairwise comparisons, craniotomy/trepanation was more frequent in POAF (81.0% vs. 63.0%; OR 2.49, 95% CI 1.18–5.45; BH-adjusted *p* = 0.036), while burr-hole/twist-drill procedures were more common in SR (28.3% vs. 12.7%; OR 0.37, 95% CI 0.15–0.87; BH-adjusted *p* = 0.036). Emergency procedures were more frequent in POAF (49.4% vs. 26.1%; OR 2.75, 95% CI 1.39–5.53; *p* = 0.002), whereas elective procedures were more common in SR. Operative time did not differ significantly between groups (*p* = 0.282). Intraoperative complication rates were similar, with no clinically meaningful difference observed.

### 4.4. Postoperative Complications

Postoperative complications are summarized in Table 8. Patients with POAF experienced a more complicated postoperative course. Pneumonia was more frequent in POAF (19.0% vs. 6.5%; OR 3.34, 95% CI 1.15–11.1; *p* = 0.018). New neurological deficits occurred more often (34.2% vs. 17.4%; OR 2.45, 95% CI 1.15–5.40; *p* = 0.014), as did seizures (12.7% vs. 3.3%; OR 4.26, 95% CI 1.05–25.0; *p* = 0.039) and delirium (13.9% vs. 4.3%; OR 3.53, 95% CI 0.99–15.9; *p* = 0.032). Deep cranial infection or meningitis occurred in 5.1% of POAF patients and in none of the SR group. Cardiogenic shock was observed more frequently in POAF (7.6% vs. 1.1%; OR 7.41, 95% CI 0.87–347.14; *p* = 0.050). Re-operation was also more common in POAF (19.0% vs. 5.4%; OR 3.72, 95% CI 1.19–13.88; *p* = 0.006). Postoperative fever occurred more frequently in POAF (23.1% vs. 4.4%; OR 6.46, 95% CI 1.99–27.54; *p* = 0.0004), with most cases attributed to urinary tract infections. Other complications were infrequent and did not differ significantly between groups. Among patients with POAF, 15 (19.0%) received anticoagulation therapy.

### 4.5. Laboratory Findings

Preoperatively, patients with POAF showed higher INR (1.212 vs. 1.051; *p* = 0.024), aPTT (34.1 vs. 31.4 s; *p* = 0.0066), CRP (3.61 vs. 1.61 mg/L; *p* = 0.0115), and troponin levels (4.81 vs. 1.15 ng/L; *p* = 0.028). Postoperatively, these differences persisted and increased, with higher INR (1.145 vs. 1.048; *p* = 1.38 × 10^−5^), aPTT (36.5 vs. 31.8 s; *p* = 0.0058), CRP (6.18 vs. 2.40 mg/L; *p* < 0.01), and NT-proBNP levels (1855 vs. 379 pg/mL; *p* = 0.004) in the POAF group. Hemoglobin and creatinine were comparable between groups.

## 5. Discussion

Across the literature, POAF has consistently been associated with higher rates of stroke, increased mortality, and prolonged hospitalization [1,2,3,4]. Notably, most studies, reviews, and meta-analyses either combine cardiac and non-cardiac surgery or address heterogeneous surgical populations, whereas data specifically focused on neurosurgical patients remain limited. Given the observational design, our findings do not establish POAF as a causal determinant of adverse outcomes. Rather, POAF likely reflects a higher-risk perioperative phenotype characterized by greater comorbidity burden, procedural stress, and postoperative complications. Several outcomes, including cardiogenic shock and short-term mortality, were based on low event numbers and therefore yielded wide confidence intervals. These findings should be interpreted cautiously, as the magnitude of association is imprecise. Importantly, the present study does not estimate the incidence of POAF but reflects a selected subgroup of patients who underwent ECG-based evaluation.

Our study was designed to examine whether newly detected POAF represents a marker of an unfavorable postoperative course. Based on our results, its occurrence was strongly associated with adverse perioperative outcomes. In this cohort, POAF appeared to be a marker of a more vulnerable postoperative clinical phenotype. In addition, these patients were exposed to more invasive surgical approaches and a greater likelihood of urgent procedures. Gialdini et al. demonstrated in a large retrospective cohort study involving more than 24,000 patients that new-onset atrial fibrillation during hospitalization after non-cardiac surgery was associated with a higher long-term risk of ischemic stroke [1]. Butt et al. analyzed Danish nationwide registry data from 1996 to 2015 and reported that POAF after non-cardiac surgery was associated with a long-term thromboembolic risk comparable to that of patients with non-surgical, non-valvular atrial fibrillation. In that study, POAF was not a benign transient condition with lower risk, but rather a condition associated with a risk profile similar to conventional AF. However, patients with POAF were significantly less likely to receive anticoagulation therapy. This finding highlights the complexity of anticoagulation decisions in patients with POAF after non-cardiac surgery [2].

Consistent with the literature on POAF in cardiac surgery, our findings are comparable to those reported in postoperative cardiac populations. Greenberg et al., in a broad review of POAF following cardiac operations, reported that POAF is associated with older age, cardiovascular risk factors such as hypertension and coronary artery disease, and the type and complexity of surgery. They also described increased risks of stroke, reoperation for bleeding, infection, renal or respiratory failure, cardiac arrest, and cerebral complications. Moreover, POAF has been shown to increase ICU utilization, hospital treatment costs, and overall length of stay [3,4]. Our findings are consistent with these observations, as neurosurgical patients with POAF similarly exhibited higher rates of postoperative complications, longer ICU and hospital stays, and higher short-term mortality. In this regard, our data support the view that POAF is a clinically relevant complication across surgical specialties.

In our cohort, pneumonia emerged as a particularly important complication, occurring in nearly one in five POAF patients and with an approximately threefold higher risk compared with patients remaining in SR. Xiang et al. identified craniotomy as a key procedural risk factor for post-craniotomy pneumonia [14]. In our study, craniotomy was also significantly more frequent in POAF patients, emphasizing the association between invasive surgical exposure, pulmonary complications, and arrhythmia vulnerability. Pneumonia further increases the complexity of postoperative management in patients who have already undergone brain surgery and subsequently develop atrial fibrillation. The observed differences in surgical access likely reflect more than technical approach alone. Craniotomy/trepanation generally represents a more invasive operative exposure associated with greater physiological stress and higher clinical acuity, whereas burr-hole/twist-drill procedures are typically less invasive. Therefore, these findings likely reflect underlying case mix and procedural complexity rather than access route alone.

Postoperative neurological complications are clinically important outcomes in this setting. New deficits, seizures, and delirium were notably more frequent in the POAF group. Their clustering with atrial fibrillation points to an interaction between systemic stress and brain vulnerability. In addition, patients with POAF demonstrated less favorable functional trajectories, with a lower likelihood of returning directly home and a greater need for rehabilitation or step-down care. The brain–heart connection provides a useful framework for interpreting these observations. Oppenheimer described the insular cortex as a central structure in cardiac autonomic regulation. Based on experimental data, the insula exerts lateralized control over autonomic tone, with right-sided lesions predisposing to sympathetic overactivation and tachyarrhythmias, whereas left-sided lesions may favor parasympathetic dominance and bradyarrhythmia [10]. Clinical studies also support a bidirectional relationship between the brain and the heart that is relevant to our findings. Battaglini et al. demonstrated that major causes of mortality after acute ischemic stroke are often cardio-related, including myocardial dysfunction, ventricular dysfunction, hemodynamic instability, cardiac arrest, and arrhythmias associated with poor outcomes and death [11]. In addition, systemic stress may increase cardiac vulnerability. Nef et al. showed that neurohumoral activation can trigger transient cardiac dysfunction in Takotsubo cardiomyopathy [21]. Goldberger et al. further emphasized that autonomic imbalance, inflammation, and neurohormonal signaling may act together to sustain arrhythmias and myocardial injury [13]. Taken together, basic, translational, and clinical research suggests that perioperative stress, autonomic imbalance, atrial remodeling, and neurohormonal activation may foster conditions leading to atrial fibrillation [10,12,13,21]. Critical illness and systemic inflammation are likewise associated with AF through autonomic imbalance, myocardial strain, and inflammatory signaling, consistent with observations in sepsis and ICU cohorts [5,12,13,22,23].

The biomarker profile observed in patients with POAF likely reflects multiple overlapping pathophysiological mechanisms. Elevated CRP levels are consistent with systemic inflammatory activation, which is common in the perioperative setting and may contribute to atrial vulnerability. In contrast, increased troponin and NT-proBNP levels may indicate myocardial strain, perioperative hemodynamic stress, or underlying cardiovascular disease. Importantly, because of the retrospective design and the limited granularity of preoperative cardiac assessment, it is not possible to distinguish whether these biomarker elevations reflect pre-existing cardiac pathology, acute perioperative stress responses, or a combination of both. Therefore, these findings should be interpreted as markers of a higher-risk systemic and cardiocirculatory state rather than as specific mechanistic indicators [5,11,12].

In clinical practice, the combination of ECG and laboratory abnormalities should prompt heightened vigilance, with a low threshold for additional imaging, infection screening, and hemodynamic optimization. Rhythm monitoring beyond routine spot ECGs may be particularly relevant in vulnerable patients characterized by older age, higher rates of hypertension, coronary artery disease, and obesity. Considering POAF as a marker of an unfavorable postoperative course, extended rhythm monitoring may merit further evaluation in selected higher-risk neurosurgical subgroups, particularly in prospective studies. Evidence from cryptogenic stroke populations, in which prolonged monitoring substantially increases AF detection, supports the rationale for such future evaluation [16,17,18,19,20].

Antithrombotic management in this setting remains a significant clinical challenge. Current guidelines recommend anticoagulation based on CHA_2_DS_2_-VASc risk irrespective of AF duration [6,7,8,9]. Randomized evidence from the ELAN trial in acute ischemic stroke suggests that earlier anticoagulation can be initiated safely within imaging-guided algorithms; however, neurosurgical patients were excluded, which limits the direct applicability of these findings [9,15]. The neurosurgical setting therefore requires particular caution, with careful balancing of thromboembolic and hemorrhagic risks. In our POAF cohort, decisions regarding anticoagulation were particularly challenging. One patient in the POAF cohort developed intracranial bleeding under anticoagulation among 14 patients who received anticoagulation, whereas three patients without anticoagulation experienced thromboembolic events. The balance between thromboembolic and hemorrhagic risks in this patient population remains uncertain. Perioperative antithrombotic strategy in neurosurgery is further complicated by choices regarding venous thromboembolism prophylaxis, with low-molecular-weight and unfractionated heparin demonstrating different efficacy and safety profiles in cranial surgery populations. This highlights the importance of tailoring strategies to individual patient management [14,24].

Anticoagulation decisions in this cohort were individualized and based on interdisciplinary assessment, balancing thromboembolic risk against the high risk of postoperative intracranial bleeding. The timing of anticoagulation initiation was not standardized and depended on clinical factors including surgical indication, postoperative imaging findings, neurological status, and overall patient stability. In general, initiation of anticoagulation was considered only after sufficient postoperative hemostasis had been ensured and in the absence of radiological signs of bleeding complications.

This approach reflects real-world clinical practice in neurosurgical patients, where decisions must be carefully tailored and frequently reassessed. At the same time, it underscores the current lack of evidence-based recommendations regarding optimal timing and patient selection for anticoagulation in this high-risk population. It should also be noted that most AF anticoagulation trials excluded neurosurgical patients because of bleeding concerns, resulting in limited direct evidence to guide management in this setting [15].

### Limitations

This study has several limitations. First, its retrospective single-center design limits causal inference and generalizability. Second, selection bias is likely, as only patients with both pre- and post-operative ECGs were included, and postoperative ECG acquisition was clinically driven rather than protocolized. Accordingly, the analyzed cohort likely represents a higher-risk subgroup of neurosurgical patients rather than the overall surgical population. Third, AF detection relied on clinically obtained ECGs, while continuous telemetry was not uniformly applied, which may have led to underdetection of transient or asymptomatic episodes. Detection bias and possible misclassification therefore cannot be excluded. In addition, detailed adjudication of AF episode duration, rhythm burden, and distinction from other supraventricular arrhythmias was not uniformly available in this retrospective dataset. Fourth, the sample size was modest, and several outcomes were infrequent, resulting in wide confidence intervals and limited precision. Several estimates, particularly for low-frequency outcomes such as cardiogenic shock and 30-day mortality, should therefore be interpreted cautiously. Fifth, due to limited event numbers, multivariable regression analyses were not performed, and residual confounding cannot be excluded. In particular, confounding by age, comorbidity burden, procedural complexity, and clinical acuity remains possible. Therefore, the findings should be considered exploratory and hypothesis-generating.

Nonetheless, the consistency of the observed associations across pulmonary, neurological, and systemic domains, together with their biological plausibility, supports the clinical relevance of the findings. Our aim is to draw attention to this underrecognized clinical challenge in neurosurgical patients. Confirmation and refinement of these observations will require prospective studies with standardized perioperative rhythm monitoring, predefined adjudication of arrhythmic events, and sufficiently powered analyses to allow adjusted modeling.

## 6. Conclusions

In conclusion, newly detected postoperative atrial fibrillation (POAF) is associated with a higher-risk postoperative course in neurosurgical patients. Compared with patients who remained in sinus rhythm, POAF clustered with older age and hypertension, more frequent craniotomy and urgent procedures, higher rates of pneumonia and neurological complications, prolonged ICU and hospital stay, lower likelihood of discharge home, and higher short-term mortality.

The observed biomarker profile (CRP, troponin, NT-proBNP) is consistent with a state of perioperative stress and myocardial strain within a brain–heart interaction framework. In this context, POAF should be considered a clinically relevant marker of an adverse postoperative phenotype rather than a coincidental finding.

These findings suggest that enhanced rhythm surveillance and structured perioperative management strategies may warrant further evaluation in selected higher-risk neurosurgical patients. Similarly, individualized antithrombotic management within a multidisciplinary framework remains essential, given the need to balance thromboembolic and hemorrhagic risks in this population.

Finally, our results highlight an important clinical gap. Confirmation and refinement of these findings will require prospective studies with standardized perioperative monitoring and predefined care pathways to determine whether targeted detection and management strategies can improve outcomes in this high-risk population.

## Figures and Tables

**Table 1:** Baseline characteristics of the study population stratified by the occurrence of postoperative atrial fibrillation (POAF). Continuous variables are presented as mean ± standard deviation, and categorical variables as number (percentage). POAF: postoperative atrial fibrillation; BMI: body mass index.

Baseline Characteristics			
Variable	POAF (n = 79)	NoPOAF (n = 92)	*p*-Value
Age (years)	70.53 ± 14.68	63.87 ± 14.83	0.0037
Height (cm)	168.87 ± 14.55	171.65 ± 8.87	0.173
Weight (kg)	78.50 ± 20.25	77.95 ± 16.50	0.857
BMI (kg/m^2^)	27.11 ± 6.00	25.97 ± 4.91	0.220
Sex (m/f)	42 (53.2%)/37 (46.8%)	40 (43.5%)/52 (56.5%)	0.267
30-day mortality, n (%)	7 (8.9%)	1 (1.1%)	0.0251

**Table 2:** Pre-existing comorbidities of the study population stratified by the occurrence of postoperative atrial fibrillation (POAF). Data are presented as number (percentage). Odds ratios (OR) with 95% confidence intervals (CI) are shown. COPD: chronic obstructive pulmonary disease.

Comorbidities				
Condition	POAF (n/%)	NoPOAF (n/%)	OR (95% CI)	*p*-Value
Hypertension	58 (73.4%)	37 (40.2%)	4.07 (2.05–8.32)	<0.0001
Coronary artery disease	18 (22.8%)	9 (9.8%)	2.71 (1.07–7.33)	0.022
Obesity	18 (22.8%)	13 (14.1%)	1.79 (0.76–4.30)	0.166
Heart failure	9 (11.4%)	3 (3.3%)	3.79 (0.90–22.55)	0.068
Diabetes mellitus	28 (35.4%)	22 (23.9%)	1.74 (0.85–3.59)	0.129
COPD	11 (13.9%)	7 (7.6%)	1.96 (0.65–6.29)	0.216
Dyslipidemia	22 (27.8%)	18 (19.6%)	1.58 (0.73–3.46)	0.211
Prior myocardial infarction	11 (13.9%)	7 (7.6%)	1.96 (0.65–6.29)	0.216
Peripheral arterial disease	6 (7.6%)	4 (4.3%)	1.80 (0.41–9.02)	0.516

**Table 3:** Resource utilization of the study population stratified by the occurrence of postoperative atrial fibrillation (POAF). Continuous variables are presented as mean ± standard deviation, with 95% confidence intervals (CI). ICU: intensive care unit.

Ressource Utilization				
Metric	POAF (Mean ± SD)	NoPOAF (Mean ± SD)	95% CI	*p*-Value
ICU length of stay (days)	3.84 ± 5.31	2.16 ± 3.13	0.32–3.02	0.0155
Total hospital stay (days)	14.24 ± 10.02	10.86 ± 7.72	0.64–6.13	0.0159

**Table 4:** Discharge destination of the study population stratified by the occurrence of postoperative atrial fibrillation (POAF). Data are presented as number (percentage). Global differences were assessed using Fisher’s exact test, followed by pairwise comparisons with Benjamini–Hochberg (BH) adjustment. Odds ratios (OR) with 95% confidence intervals (CI) are shown. SR: sinus rhythm.

Discharge Destination (Global + Pairwise)					
Global Fisher’s Exact: *p* = 0.0288					
Destination	POAF n (%)	SR n (%)	OR (95% CI)	*p* (raw)	*p* (BH)
Home	33 (45.8%)	61 (66.3%)	0.43 (0.22–0.85)	0.0109	0.0436
Rehabilitation	15 (20.8%)	9 (9.8%)	2.41 (0.92–6.72)	0.0733	0.217
Nursing facility	1 (1.4%)	1 (1.1%)	1.28 (0.02–101.62)	1.000	1.000
Oncologic/Geriatric ward	23 (31.9%)	19 (20.7%)	1.80 (0.84–3.90)	0.108	0.217

**Table 5:** Primary diagnosis of the study population stratified by the occurrence of postoperative atrial fibrillation (POAF). Data are presented as number (percentage). Global differences were assessed using Fisher’s exact test, followed by pairwise comparisons with Benjamini–Hochberg (BH) adjustment. Odds ratios (OR) with 95% confidence intervals (CI) are shown. CSF: cerebrospinal fluid.

Primary Diagnosis (Global + Pairwise)					
Global Fisher’s Exact: *p* = 0.018					
Category	POAF n (%)	NoPOAF n (%)	OR (95% CI)	*p* (raw)	*p* (BH)
Vascular	25 (31.6%)	18 (19.6%)	1.90 (0.89–4.09)	0.079	0.291
Neoplastic (Tumors)	41 (51.9%)	59 (64.1%)	0.61 (0.31–1.17)	0.121	0.291
Traumatic	10 (12.7%)	6 (6.5%)	2.07 (0.64–7.29)	0.195	0.292
Hydrocephalus/CSF disorders	1 (1.3%)	6 (6.5%)	0.19 (0.00–1.58)	0.125	0.291
Infectious/Inflammatory	0 (0.0%)	3 (3.3%)	0.00 (0.00–2.80)	0.250	0.292
Epilepsy surgery	2 (2.5%)	0 (0.0%)	Inf (0.22–Inf)	0.212	0.292

**Table 6:** Surgical access of the study population stratified by the occurrence of postoperative atrial fibrillation (POAF). Data are presented as number (percentage). Global differences were assessed using Fisher’s exact test, followed by pairwise comparisons with Benjamini–Hochberg (BH) adjustment. Odds ratios (OR) with 95% confidence intervals (CI) are shown. CSF: cerebrospinal fluid.

Surgical Access (Global + Pairwise)					
Global Fisher’s Exact: *p* = 0.079					
Access	POAF n (%)	NoPOAF n (%)	OR (95% CI)	*p* (raw)	*p* (BH)
Craniotomy/Trepanation	64 (81.0%)	58 (63.0%)	2.49 (1.18–5.45)	0.011	0.036
Burr-hole/Twist-drill	10 (12.7%)	26 (28.3%)	0.37 (0.15–0.87)	0.015	0.036
CSF/Shunt procedures	1 (1.3%)	2 (2.2%)	0.58 (0.01–11.31)	1.000	1.000
Reconstructive procedures	3 (3.8%)	4 (4.3%)	0.87 (0.12–5.31)	1.000	1.000
Device-related procedures	1 (1.3%)	2 (2.2%)	0.58 (0.01–11.31)	1.000	1.000

**Table 7:** Surgical urgency (emergency vs. elective) of the study population stratified by the occurrence of postoperative atrial fibrillation (POAF). Data are presented as number (percentage). The association between emergency and elective procedures is expressed as odds ratio (OR) with 95% confidence interval (CI). Statistical comparisons were performed using Fisher’s exact test and χ^2^ test with Yates’ correction. SR: sinus rhythm.

Urgency (Emergency vs. Elective)		
Status	POAF n (%)	SR n (%)
Emergency	39 (49.4%)	24 (26.1%)
Elective	40 (50.6%)	68 (73.9%)
**Association (Emergency vs. elective): OR 2.75 (95% CI 1.39–5.53), Fisher *p* = 0.00241 (χ^2^ Yates *p* = 0.00281)**		

**Table 8:** Postoperative complications of the study population stratified by the occurrence of postoperative atrial fibrillation (POAF). Data are presented as number (percentage). Odds ratios (OR) with 95% confidence intervals (CI) are shown where applicable. Statistical comparisons were performed using χ^2^ or Fisher’s exact test, as appropriate. SR: sinus rhythm.

Postoperative Complications				
Complication	POAF (n = 79)	SR (n = 92)	OR (95% CI)	*p*-Value
Pneumonia	15 (19.0%)	6 (6.5%)	3.34 (1.15–11.1)	0.018
New neurological deficit	27 (34.2%)	16 (17.4%)	2.45 (1.15–5.40)	0.014
Seizures	10 (12.7%)	3 (3.3%)	4.26 (1.05–25.0)	0.039
Delirium	11 (13.9%)	4 (4.3%)	3.53 (0.99–15.9)	0.032
Deep cranial infection/meningitis	4 (5.1%)	0 (0%)	x	0.044
Cardiogenic shock	6 (7.6%)	1 (1.1%)	7.41 (0.87–347.14)	0.050
Re-operation	15 (19.0%)	5 (5.4%)	3.72 (1.19–13.88)	0.006
Postoperative fever	18 (23.1%)	4 (4.4%)	6.46 (1.99–27.54)	0.0004

## Data Availability

The data supporting the findings of this study are available from the corresponding author upon reasonable request.
